# Antibody-Targeted TNFRSF Activation for Cancer Immunotherapy: The Role of FcγRIIB Cross-Linking

**DOI:** 10.3389/fphar.2022.924197

**Published:** 2022-07-05

**Authors:** Luyan Liu, Yi Wu, Kaiyan Ye, Meichun Cai, Guanglei Zhuang, Jieyi Wang

**Affiliations:** ^1^ Lyvgen Biopharma, Shanghai, China; ^2^ State Key Laboratory of Oncogenes and Related Genes, Department of Obstetrics and Gynecology, Ren Ji Hospital, Shanghai Cancer Institute, Shanghai Jiao Tong University School of Medicine, Shanghai, China; ^3^ Shanghai Key Laboratory of Gynecologic Oncology, Ren Ji Hospital, Shanghai Jiao Tong University School of Medicine, Shanghai, China

**Keywords:** FcγRIIB, TNFRSF, CD40, CD137 (4-1BB), agonist, immunotherapy, cancer, cross-linking

## Abstract

Co-stimulation signaling in various types of immune cells modulates immune responses in physiology and disease. Tumor necrosis factor receptor superfamily (TNFRSF) members such as CD40, OX40 and CD137/4-1BB are expressed on myeloid cells and/or lymphocytes, and they regulate antigen presentation and adaptive immune activities. TNFRSF agonistic antibodies have been evaluated extensively in preclinical models, and the robust antitumor immune responses and efficacy have encouraged continued clinical investigations for the last two decades. However, balancing the toxicities and efficacy of TNFRSF agonistic antibodies remains a major challenge in the clinical development. Insights into the co-stimulation signaling biology, antibody structural roles and their functionality in immuno-oncology are guiding new advancement of this field. Leveraging the interactions between antibodies and the inhibitory Fc receptor FcγRIIB to optimize co-stimulation agonistic activities dependent on FcγRIIB cross-linking selectively in tumor microenvironment represents the current frontier, which also includes cross-linking through tumor antigen binding with bispecific antibodies. In this review, we will summarize the immunological roles of TNFRSF members and current clinical studies of TNFRSF agonistic antibodies. We will also cover the contribution of different IgG structure domains to these agonistic activities, with a focus on the role of FcγRIIB in TNFRSF cross-linking and clustering bridged by agonistic antibodies. We will review and discuss several Fc-engineering approaches to optimize Fc binding ability to FcγRIIB in the context of proper Fab and the epitope, including a cross-linking antibody (xLinkAb) model and its application in developing TNFRSF agonistic antibodies with improved efficacy and safety for cancer immunotherapy.

## 1 Introduction

Immunotherapy has become an important cancer treatment option, and the antagonistic antibodies of immune checkpoint inhibitors PD-(L)1 have led a cancer treatment revolution in the last decade. Many studies have revealed the roles of adaptive immunities in tumor elimination and the mechanisms by which cancers evade immune response (Wei, Duffy et al. 2018, Morad, Helmink et al. 2021). CD8 T-cell dysfunction was identified as one mechanism of tumor escape, sometimes caused by overactivation of immune checkpoint receptors, such as PD-1. Since the response to PD-(L)1 treatment varies among patients and cancer types, with a limited response rate of around 10%–30%, it is important to identify new immuno-therapeutic targets and optimize the corresponding drug design with improved therapeutic efficacy and safety.

Co-stimulatory receptors have been considered as important as and complementary to the checkpoint inhibitors to promote immune antitumor activities. Many tumor necrosis factor (TNF) receptor superfamily (TNFRSF) members have been identified as the co-stimulatory receptors in antitumor immunity (Croft et al. 2013). For example, the TNFRSF members CD137 (4-1BB) and CD40 showed their potential as immunotherapy targets by stimulating the proliferation and cytotoxic activities of tumor-reactive CD8 T cells directly and indirectly, respectively (Jeong and Park, 2020). Effective TNFRSF agonistic antibodies may contribute to the therapeutic repertoires against cancers.

TNFRSF receptors are normally activated by molecular clustering induced by cognate cell membrane TNFSF ligands. Antibodies have bivalency in antigen binding and can induce cell membrane receptor clustering and activation. The binding epitope of the TNFRSF target is one of the key factors determining the intrinsic agonistic activities of the antibody. Antibodies are also multi-domain immunoglobulins possessing Fc receptor interaction in addition to antigen binding functionality. Thus, the agonistic activity of an antibody can vary drastically in different environments resulting from potential multiple molecular interactions. In fact, Fc and Fc gamma receptor (FcγR) interaction has been identified as the most dominant factor in determining overall agonistic activities of an antibody. Binding to FcγRs *via* Fc provides the antibody an opportunity for multivalent interactions beyond its antigen target. Thus, Fc/FcγR interaction can facilitate target receptor cross-linking and clustering by TNFRSF agonistic antibodies (Medler et al. 2019). As receptor molecule clustering is the fundamental mechanism for TNFRSF members to trigger their downstream signaling, Fc/FcγR mediated cross-linking can dramatically affect the agonistic activity of TNFRSF antibodies, generating super TNFRSF agonistic antibodies in certain cases.

Here, we review recent advances in our understanding of agonistic antibodies targeting TNFRSF in cancers. We focus on the structural and functional correlations of these TNFRSF agonistic antibodies, more specifically the impact of Fc-gamma receptor IIB (FcγRIIB) on their antitumor activities and safety. We will discuss the principles of how FcγRIIB-dependent TNFRSF agonistic antibodies can be engineered to induce receptor clustering and activation optimally in the tumor microenvironment while minimizing systemic toxicity.

## 2 TNFSF-TNFRSF System

### 2.1 TNFSF-TNFRSF Expression Profile and Biological Function

**FIGURE 1:**
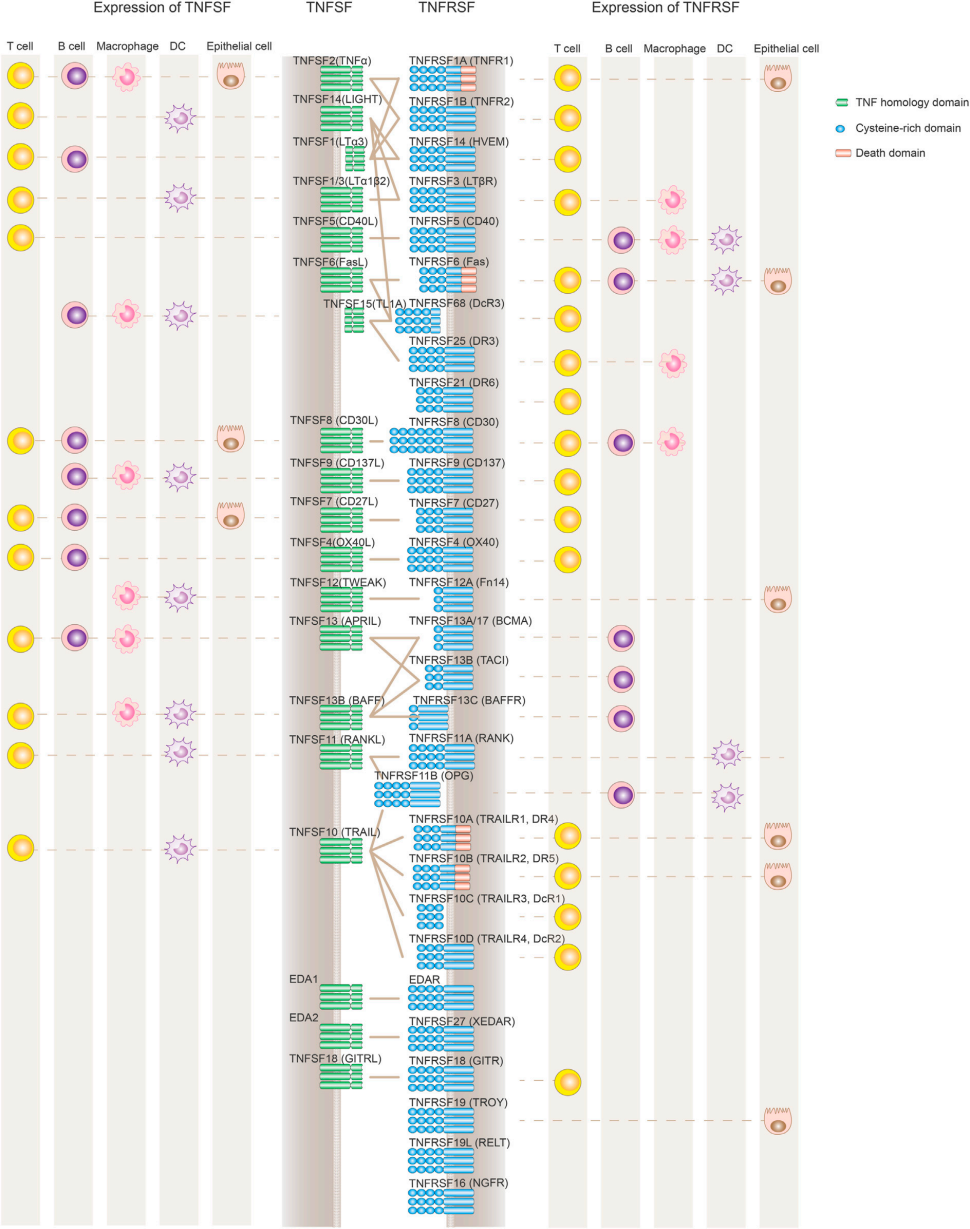
Expression and interaction of TNFSF and TNFRSF members. TNFRSF members (depicted in blue on the right) contain variable numbers of cysteine-rich domains (CRD) in their ligand-binding extracellular regions. TNFSF ligands (left side shown in green) are active primarily as non-covalently associated homotrimers or homodimers to facilitate the formation of TNFRSF trimer clustering and its downstream signaling activation. TNFRSF members regulate the immune system mainly through either stimulating cell proliferation and maturation or promoting apoptotic cell death via a death domain. Also depicted are the primary cell types expressing TNFRSF and TNFSF, although the list is not comprehensive to represent the complex expression profile of each molecule, and some cell populations such as NK cells or monocytes are not included. Both TNFSF and TNFRSF members are widely and dynamically expressed in different immune cell populations.

The tumor necrosis factor superfamily (TNFSF) and their receptor superfamily (TNFRSF), composed of 19 ligands and 29 receptors respectively, play diversified roles in inflammation, apoptosis, proliferation and morphogenesis (Figure 1; Table 1) (Dostert et al. 2019). The genes of TNFSF and TNFRSF are clustered in several distinct genetic regions within the major histocompatibility complex (MHC). The human MHC is a large genetic region containing a dense proportion of genes implicated in immunity. Phylogenetic analyses clearly indicate that TNFSF and TNFRSF emerged and diverged from the common ancestors within proto-MHC region before the appearance of vertebrates. TNFSF and TNFRSF co-evolve together with the adaptive immune system (Collette et al., 2003, Glenney and Wiens, 2007, Wiens and Glenney, 2011). That evolutionary history might explain the highly conserved structural and functional properties among TNFSF and TNFRSF members. They have an important role in adaptive immunity and control of immune homeostasis. Structurally, they share a similar ligand-receptor trimeric structure for signaling activation. Functionally, TNFRSF members show some overlapping expression profiles and biological functions such as promoting the proliferation of T and B cells or maturation of dendritic cells and macrophages. At the same time, each TNFSF or TNFRSF member is assumed to have its distinct spatiotemporal expression profile, signaling network, and functional impact on the immune system.

**TABLE 1:** Tumor necrosis factor superfamily ligands and receptors and their functions.

Ligand (alternative Name)	Ligand KO mice phenotype	Receptor (alternative Name)	Receptor KO mice phenotype	Ligand or receptor human disease association
TNFSF1 (TNF-β, LT-α)	Defects in secondary lymphoid organ development; disorganized splenic microarchitecture Banks et al. (1995)	TNFRSF1A (TNFR1, DR1)	Resistant to low levels of LPS; increased susceptibility to *Listeria* monocytogenes infection Rothe et al. (1993); impaired oval cell proliferation; reduction of tumorigenesis Knight et al. (2000)	Cerebral infarction; TNFR1-associated periodic syndrome McDermott et al. (1999); TRAPS associated with SLE Ida et al. (2004); Crohn disease Waschke et al. (2005); multiple sclerosis Gregory et al. (2012)
		TNFRSF1B (TNFR2)	Increased sensitivity to bacterial pathogens; decreased sensitivity to LPS; reduced antigen-induced T-cell apoptosis Locksley et al. (2001)	Crohn’s Disease Sashio et al. (2002); Waschke et al. (2005)
TNFSF2 (TNF-α)	No phenotypic abnormalities in LN; lack splenic primary B-cell follicles; disorganized FDC networks and germinal centers Pasparakis et al. (1996); resistant to skin carcinogenesis	TNFRSF1A (TNFR1, DR1)	Resistant to low levels of LPS; increased susceptibility to *Listeria* monocytogenes infection Rothe et al. (1993); impaired oval cell proliferation; reduction of tumorigenesis Knight et al. (2000)	Cerebral infarction Um et al. (2003); TNFR1-associated periodic syndrome McDermott et al. (1999); TRAPS associated with SLE Ida et al. (2004); Crohn disease Waschke et al. (2005)
		TNFRSF1B (TNFR2)	Increased sensitivity to bacterial pathogens; decreased sensitivity to LPS; reduced antigen-induced T-cell apoptosis Locksley et al. (2001)	Crohn’s Disease Waschke et al. (2005)
TNFSF3 (LT-β)	Defects in organogenesis of the lymphoid system; lymphocytosis in the circulation and peritoneal cavity; lymphocytic infiltrations in lungs and liver Alimzhanov et al. (1997)	TNFRSF3 (LT-βR)	Absence of LN, PP; defective GC formation Fütterer et al. (1998); Locksley et al. (2001); lack the CD11b+CD8^−^ DC subset	Not reported
TNFSF4 (OX40L, CD252, gp34)	Defective dendritic cell-dependent T-cell response Locksley et al. (2001); not protected against L. Major infection Ishii et al. (2003)	TNFRSF4 (OX40, CD134)	Defective T-cell responses Locksley et al. (2001); Th2 is impaired and competent in B cell immunity Akiba et al. (2000)	primary immunodeficiency, Kaposi sarcoma Byun et al. (2013); systemic sclerosis Gourh et al. (2010)
TNFSF5 (CD40L, CD154, gp39)	Decreased IgM response Xu et al. (1994); Defective T-cell and IgG responses; hyper-IgM syndrome Locksley et al. (2001); exhibit prolonged bleeding time and decreased shear-induced platelet aggregates Crow et al. (2003)	TNFRSF5 (CD40, p50)	Impaired B cell maturation Khan et al. (1997); Defective Ig class switching and GC formation causing immunodeficiency Locksley et al. (2001); succumbed to low-dose aerosol infection with *M. Tuberculosis* due to deficient IL-12 production leading to impaired priming of IFN-gamma T cell responses Ishikawa et al. (2005)	X-linked hyper-IgM syndrome Allen et al. (1993)
TNFSF6 (FasL, CD95L, Apo1L)	Impaired activation-induced T-cell death; lymphoproliferation; autoimmunity Locksley et al. (2001); causes massive lymphoproliferation and early death Karray et al. (2004)	TNFRSF6 (Fas, CD95, Apo1, DR2)	Impaired activation-induced T-cell death; lymphoproliferative syndrome; autoimmunity Adachi et al. (1996); Accumulation of autoreactive B cells in T cell-rich zones; production of autoantibodies Rieux-Laucat et al. (1995); Resistant to suppression by high doses of antigen and to apoptosis in mature CD4 T cells Singer and Abbas (1994)	Generalized lymphoproliferative disease Takahashi et al. (1994)
		TNFRSF6B (DcR3)	Not reported	Not reported
TNFSF7 (CD27L, CD70)	Impaired effector CD8 T cell generation and viral clearance Munitic et al. (2013) but dispensable for the recall response to lymphocytic choriomeningitis virus	TNFRSF7 (CD27)	Defective T-cell responses Locksley et al. (2001)	Autosomal recessive lymphoproliferative syndrome-2 (LPFS2) van Montfrans et al. (2012)
TNFSF8 (CD30L, CD153)	Not reported	TNFRSF8 (CD30)	Impaired follicular GC responses; reduced recall-memory Ab responses Gaspal et al. (2005); impaired thymic negative selection and increased autoreactivity Blazar et al. (2004)	Not reported
TNFSF9 (4–1BBL)	Defective T-cell responses Locksley et al. (2001); unimpaired CTL responses to lymphocytic choriomeningitis virus (LCMV), normal skin allograft rejection, and a weaker CTL response to influenza virus DeBenedette, Wen et al. (1999)	TNFRSF9 (4–1BB, CD137, ILA)	Normal T-cell development Kwon et al. (2002); Decreased CD8 T cell number Steinhilber and Stegmeyer (1977), Increased CD4 T-cell infiltration Vinay et al. (2007); Reduced number of NK and NKT cells; resistance to LPS-induced shock syndrome Vinay et al. (2004); Increased number of myeloid progenitor and mature DCs; impaired DC function Lee et al. (2008); Reduced atherosclerosis in hyperlipidemic mice Jeon et al. (2010)	CD137 defects in lymphoma Luu et al. (2020)
TNFSF10 (TRAIL, Apo2L)	Delayed regression of retinal neovascularization Hubert et al. (2009); Defective thymocyte apoptosis and accelerated autoimmune diseases Lamhamedi-Cherradi et al. (2003)	TNFRSF10A TRAILR1, DR4, Apo2)	Not reported	Not reported
		TNFRSF10B TRAILR2, DR5)	Normal development with an enlarged thymus Finnberg et al. (2005); TRAIL-R deficiency in mice enhances lymph node metastasis without affecting primary tumor development Grosse-Wilde et al. (2008)	Head and neck squamous cell carcinoma Pan et al. (1997)
		TNFRSF10C (TRAILR3, DcR1)	Not reported	Not reported
		TNFRSF10D (TRAILR4, DcR2)	Not reported	Not reported
TNFSF11 (RANKL, TRANCE, OPGL, ODF)	Osteopetrosis; growth retardation of limbs, skull and vertebrae; chondrodysplasia Kong et al. (1999); Kim et al. (2000)	TNFRSF11A (RANK, TRANCER)	Osteopetrosis; absence of osteoclasts and LN; PP present; abnormal B-cell development Dougall et al. (1999); Li et al. (2000); Locksley et al. (2001)	Familial expansile osteolysis Hughes et al. (2000); osteoclast-poor osteopetrosis with hypogammaglobulinemia Guerrini et al. (2008)
		TNFRSF11B (OPG, OCIF)	Osteoporosis; arterial calcification Bucay et al. (1998)	Idiopathic hyperphosphatasia Cundy et al. (2002); Juvenile Paget Disease Middleton-Hardie et al. (2006)
TNFSF12 (TWEAK, Apo3L)	Overabundant natural killer (NK) cells and displayed hypersensitivity to bacterial endotoxin; developed oversized spleens with expanded memory and T helper 1 (Th1) subtype cells upon aging and mounted stronger innate and adaptive Th1-based responses against tumor challenge Maecker et al. (2005)	TNFRSF12A (Fn14, TWEAKR)	Reduced proliferative capacity; altered myotube formation Girgenrath et al. (2006); Reduced LPC numbers; attenuated inflammation; cytokine production Tirnitz-Parker et al. (2010)	primary antibody deficiency Wang et al. (2013)
TNFSF13 (APRIL, TALL-2, TRDL-1)	Normal immune system development Varfolomeev et al. (2004); Impaired IgA class switching Castigli et al. (2004)	TNFRSF13A (BCMA)	Reduction in long-lived igg-producing PC, but normal B-cell development and humoral responses Xu and Lam (2001)	Not reported
		TNFRSF13B (TACI)	Increased B-cell accumulation; splenomegaly Yan et al. (2001b)	Common variable immunodeficiency Salzer et al. (2005); Castigli et al. (2005)
TNFSF13B (BAFF, BLYS, THANK)	Impaired B-cell maturation (86); Low Ig serum levels; block in B-cell development at the T1 stage; absence of T2, mantle and follicular zone B cells in the LN and spleen Schiemann et al. (2001)	TNFRSF13B (TACI)	Increased B-cell accumulation; splenomegaly Yan et al. (2001b)	Common variable immunodeficiency Salzer et al. (2005); Castigli et al. (2005)
		TNFRSF13C (BAFFR)	Reduced late transitional and follicular B-cell numbers; devoid of marginal zone B cells; reduced CD21 and CD23 surface expression Yan et al. (2001a); Rickert et al. (2011)	Common variable immunodeficiency Warnatz et al. (2009)
		TNFRSF17 (BCMA)	Reduction in long-lived IgG-producing PC, but normal B-cell development and humoral responses Xu and Lam (2001)	Not reported
TNFSF14 (LIGHT, HVEML, LT-γ)	No significant abnormalities in the development of lymphoid organs and lymphocytes Ward-Kavanagh et al. (2016); defects in costimulatory t cell activation and cooperation with lymphotoxin beta in mesenteric lymph node genesis Scheu et al. (2002)	TNFRSF14 (LIGHTR, HVEM)	No significant abnormalities in the development of lymphoid organs and lymphocytes Ward-Kavanagh et al. (2016)	Not reported
		TNFRSF3 (LT-βR)	Not reported	Not reported
TNFSF15 (TL1A, VEGI)	Reduced capacity in supporting Th17 differentiation and proliferation Pappu et al. (2008)	TNFRSF25 (DR3)	Impaired negative selection and anti-CD3-induced apoptosis Wang et al. (2001)	Not reported
		TNFRSF6B (DcR3)	Not reported	Not reported
TNFSF18 (GITRL)	Not reported	TNFRSF18 (GITR, AITR)	Abolished anti-CD3-induced T-cell activation Stephens et al. (2004); normal development of the lymphoid organ, no effect on antibody responses Teodorovic et al. (2012)	Not reported
EDA-A1	Ectodermal dysplasias Monreal et al. (1998)	EDAR	Abnormal tooth, hair and sweat gland formation Locksley et al. (2001)	Ectodermal dysplasias Monreal et al. (1998); Hypohidrotic ectodermal dysplasia Shimomura et al. (2004)
EDA-A2	Impaired development of hair, eccrine sweat glands, and teeth Schneider et al. (2001); Multifocal myodegeneration Newton et al. (2004)	TNFRSF27 (XEDAR)	No different than wild-type littermates Newton et al. (2004)	Not reported
To be identified	Not reported	TNFRSF19 (TROY, TAJ)	No apparent defects in skin appendages Pispa et al. (2008)	Not reported
To be identified	Not reported	TNFRSF19L (RELT)	Not reported	Not reported
Amyloid polypeptide (APP) (not a TNFSF member)	Not reported	TNFRSF21 (DR6)	Enhanced CD4 T-cell expansion and Th2 differentiation; enhanced splenic GC formation, impaired JNK activity; T-cell differentiation Zhao et al. (2001); CD4 T-cell proliferation; Th differentiation Liu et al. (2001)	Not reported
To be identified	Not reported	TNFRSF16 (NGFR, CD271)	Decreased sensory neuron innervation; impaired heat sensitivity Lee et al. (1992)	Not reported

Although there is still a lot to be learned about the nuanced divergence among the TNFSF and TNFRSF members, our knowledge has been enriched gradually during the last three decades (Aggarwal et al., 2012, Croft and Siegel, 2017, Dostert et al., 2019) (Table 1). From mouse gene knock-out studies and human genetics analyses, TNFRSF and TNFSF members have been proven to play critical roles in immune system development and immune modulation. In the case of the stimulation of T cell antitumor activities, the known T cell co-stimulators including CD27, CD137 and OX40 were demonstrated to have distinct effects. For example, CD27 and HVEM expressed on resting T cells function early after the initial activation of T cells, while OX40 and CD137 signals on T cells are induced at a later stage after antigen-activation, and OX40 and CD137 showed distinct preferential effects on CD4 and CD8 T cells respectively (Watts, 2005). In another experiment to characterize the impact of CD27, CD137, OX40, and GITR on the CD8 T-cell cytokine response, although both CD137 and CD27 increased the sensitivity of the stimulation, only CD137 was able to prolong the response duration and had the strongest amplification effect on cytokine production. In contrast, GITR and OX40 had almost no effect shown in this study (Nguyen et al., 2021). In an *ex vivo* tumor-infiltrating lymphocytes (TILs) stimulation assay with autologous tumor cells, CD137+ TILs exhibited the greatest frequency among the effector cells expressing IFN-γ, TNF-α, Granzyme B, perforin, and IL-2, compared to OX40+, PD-1+, CD25^+^, and CD69^+^ TILs, suggesting that CD137+ TILs might have the most effective antitumor activities.

### 2.2 TNSRSF Signaling Activation Driven by Receptor Clustering

**FIGURE 2:**
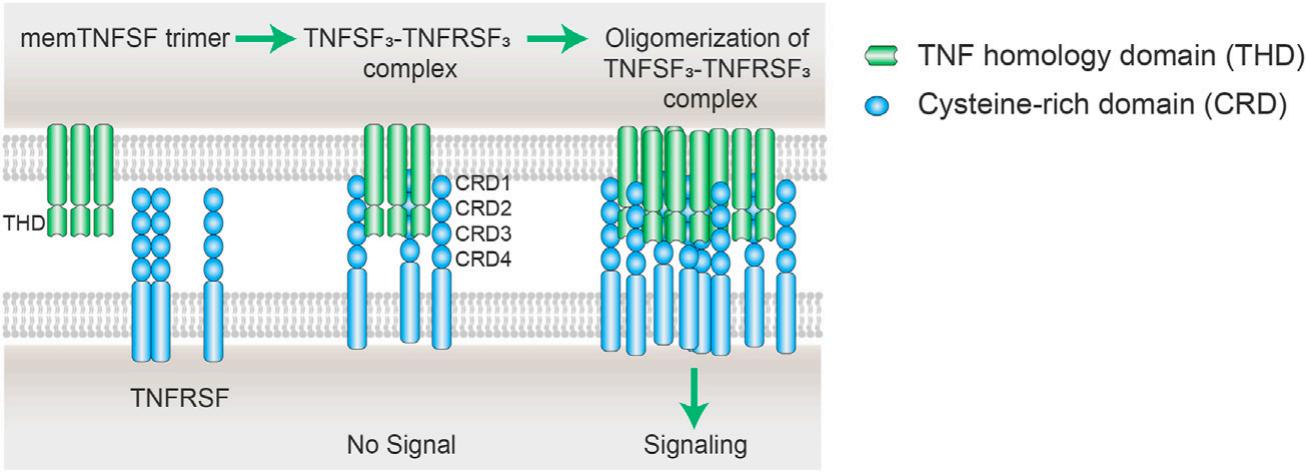
Scheme of oligomerization of TNFSF_3_–TNFRSF_3_ complexes. The cell membrane TNFSF (mem TNFSF, often associated as trimer) binds to TNFRSF *via* the interactions between the TNF homology domain (THD) and cysteine rich domain (CRD), resulting in the formation of TNFSF_3_-TNFRSF_3_ complex. However, the minimum TNFSF-TNFRSF complex is insufficient to trigger signaling of some TNFRSF members including CD40, CD137, OX40, and GITR. Secondary interaction of the initial trimeric complex leads to oligomerization of the ligand-receptor complexes. The clustering of multiple TNFRSFs is necessary to activate receptor intracellular signaling pathways.

TNFRSF members are naturally activated by their corresponding TNFSF ligands in a highly regulated manner. As above mentioned above, the structural principles of TNFRSF-TNFSF interactions and downstream signaling activation are well conserved among the TNFRSF and TNFSF members. TNFRSF receptors are type 1 transmembrane proteins that adopt elongated structures containing mainly 3-4 highly conserved “cysteine-rich domains” (CRDs). TNFSF ligands are type II transmembrane proteins non-covalently linked as homotrimer or homodimers via a conserved carboxy-terminal homology domain called the TNF homology domain (THD). TNFSF trimer mainly binds to the 2nd and 3rd CRDs (CRD 2–3) of TNFRSF members and facilitates the formation of an active TNFSF_3_–TNFRSF_3_ complex (Figure 2) (Banner et al., 1993; Locksley et al., 2001; Compaan and Hymowitz, 2006). This complex is the minimal unit for TNFRSF signaling activation. In fact, a significant fraction of TNFRSF members need further oligomerization of two or more TNFSF_3_–TNFRSF_3_ complexes to be able to fully stimulate TNFRSF downstream signaling cascades (Figure 2) (Wajant, 2015). In natural physiological conditions, the ligands of this category of TNFRSF work as membrane- bound, while soluble TNFSF does not show secondary receptor clustering ability. The molecular and cellular regulation mechanisms of TNFSF and TNFRSF enable a precise control of TNFRSF signaling with spatial-temporal specificity.

## 3 Therapeutic Potential of TNFRSF Agonistic Antibodies

Many TNFRSF members have been evaluated as potential novel immunotherapy targets, given their role in activation, clonal expansion, and survival of immune cells including cytotoxic T cells, the same population regulated by PD-1. In fact, the interest of targeting TNFRSF in medicine emerged by the end of 1990s, even earlier than the development of PD-1 antagonists. Besides T cell co-stimulators such as CD137, CD27, OX40, GITR or TNFR2, for antigen presentation and innate immune activation, CD40 is also an important target for cancer immunotherapy. Nowadays, there are dozens if not hundreds of TNFRSF agonist candidates in the format of monoclonal or multi-specific antibodies being tested in clinical trials. They are being investigated or developed as monotherapy or in combination with immune checkpoint inhibitors, such as anti-CTLA4 or anti PD-1/PD-L1 antibodies, or standard treatment like chemotherapy. The clinical development status of representative TNFRSF agonistic antibodies (Croft et al., 2013; Assal et al. 2015; Burugu et al., 2018; Lee, 2021) are summarized in Table 2.

**TABLE 2:** Clinical studies of anti-TNFRSF agonistic antibodies for cancer treatment, alone or in combination.

Target	Agent	Molecule	Clinical Status	Key Findings	NCT number
TNFRSF4 (OX40)	Ivuxolimab (PF-04518600)	IgG2	II (FIH 2015)	• No DLTs (0.01 to 10 mg/kg)	NCT02315066
				• Monotherapy: ORR 4% Diab et al. (2016); Diab et al. (2022)	
	Tavolimab (MEDI-0562)	IgG1	I (FIH 2015)	• No DLTs (0.03 to 10 mg/kg)	NCT02318394
				• Monotherapy: 2 immune-related PR Glisson et al. (2020)	
	MEDI6469	IgG1	I/II (FIH 2003)	• No MTD reached (0.1 to 2 mg/kg)	NCT02559024
				• Monotherapy: ORR 0% Curti et al. (2013)	
	GSK3174998	IgG1	I/II (FIH 2015)	• No MTD established (0.003 to 10 mg/kg)	NCT02528357
				• Monotherapy: ORR 2.2%	
				• GSK3174998/pembrolizumab; ORR 8% Postel-Vinay et al. (2020)	
	Vonlerolizumab (MOXR0916)	IgG1	I (FIH 2015)	• No DLTs (0.2–1200 mg) Hansen et al. (2016)	NCT02219724 NCT02410512
				• Vonlerolizumab/atezolizumab; ORR 4% Infante et al. (2016)	
TNFRSF5 (CD40)	Selicrelumab (CP-870893, RG-7876)	IgG2	I (FIH 2004)	• Strong agonistic activity	NCT02225002 NCT01103635 NCT02157831 NCT00607048 NCT01456585 NCT00711191
				• MTD 0.2 mg/kg, ORR 14% Vonderheide et al. (2007)	
				• Monotherapy (weekly dosing) for solid tumor: ORR 0% Rüter et al. (2010)	
				• CP-870893/chemo for solid tumors: ORR 20%–40% Beatty et al. (2013), Vonderheide et al. (2013), Nowak et al. (2015)	
	Sotigalimab (APX005M)	IgG1 mutant (S267E)	I/II (FIH 2015)	• Strong FcγRIIB binding	NCT03123783 NCT02482168 NCT03502330
				• RP2D: 0.3 mg/kg, Johnson et al. (2017)	
				• APX005M/chemo w/w.o. Nivo for pancreatic cancer: ORR 58% O'Hara et al. (2019)	
	ChiLob7/4	IgG1	I (FIH 2007)	• MTD 200 mg	NCT01561911
				• Monotherapy: ORR 0% Johnson et al. (2015)	
	SEA-CD40	IgG1 nonfucosylated	I (FIH 2015)	• Enhanced SEA-CD40/FcγRIIIa binding Gardai et al. (2016)	NCT02376699 NCT04993677
				• 5 DLTs (0.6–60 mcg/kg or 30 mcg/kg)	
				• 1 PR,10 SD, DCR 32% Grilley-Olson et al. (2018)	
	CDX-1140	IgG2	I/II (FIH 2017)	• RP2D 1.5 mg/kg Sanborn et al. (2020)	NCT03329950
	LVGN7409	IgG mutant	I (FIH 2020)	• TBD Fu et al. (2022)	NCT04635995 NCT05152212 NCT05075993
	Mitazalimab (ADC1013	IgG1	I/II (FIH 2015)	• RP2D 0.9–1.2 mg/kg Deronic et al. (2021), Enell Smith et al. (2021)	NCT02379741 NCT02829099 NCT04888312
	YH003	IgG2	I/II (FIH 2021)	• No DLTs (0.03–0.3 mg/kg)	NCT04481009
				• YH003/Toripalimab: 1PR Coward et al. (2021)	
	GEN 1042	Bispecific (CD40xCD137)	I/II (FIH 2019)	• No MTD reached (0.1–400 mg) Johnson et al. (2021)	NCT04083599
	RO7122290	Bispecific (FAPxCD40)	I (FIH 2017)	• No MTD reached (5 — 2000 mg) Melero et al. (2020)	NCT03869190
TNFRSF7 (CD27)	Varlilumab (CDX-1127)	IgG1	I/II (FIH 2011)	• No DLT (0.1–10 mg/kg)	NCT01460134
				• Monotherapy: 0%-10% Ansell et al. (2020)	
TNFRSF9 (CD137)	Urelumab (BMS-663513)	IgG4	I/II (FIH 2005)	• Strong agonistic activity	NCT01471210 NCT01775631 NCT02110082 NCT00461110 NCT00351325 NCT00309023 NCT02534506 NCT02253992
				• RP2D 0.1-0.3 mg/kg Segal et al. (2017)	
				• Monotherapy for R/R NHL: 6%–35%	
				• Urelumab/rituximab: ORR 10%–30% Timmerman et al. (2020)	
				• Urelumab/nivolumab: ORR 4.5%–50% Massarelli et al. (2016)	
	Utomilumab (PF-05082566)	IgG2	I/II (FIH 2011)	• Weak agonistic activity	NCT02179918 NCT02444793 NCT01307267
				• No DLT (0.006-10 mg/kg) Segal et al. (2018)	
				• Monotherapy; ORR 3.8% Segal et al. (2018)	
				• Utomilumab/rituximab for R/R NHL; ORR 21.2% Gopal et al. (2020)	
				• Utomilumab/pembrolizumab; ORR 26.1% Tolcher et al. (2017)	
	LVGN6051	IgG mutant	I/II (FIH 2019)	• RP2D: 4 mg/kg Qi et al. (2019), Fu et al. (2021)	NCT04130542 NCT04694781 NCT05075993
	ADG106	IgG4	I/II (FIH 2018)	• RP2D: 5 mg/kg Zhang et al. (2020)	NCT03802955 NCT03707093
	CTX-471	IgG4	I (FIH 2019)	• RP2D: 0.3, 0.6 mg/kg	NCT03881488
				• Monotherapy: 3PR, ORR 8% Compass Therapeutics (2022)	
	Cinrebafusp alfa (PRS-343)	Bispecific antibody (HER2 x CD137)	I (FIH 2017)	• Monotherapy: 1CR, 3PR	NCT03330561 NCT03650348
				• Cinrebafusp alfa / atezolizumab: 4PR Ku et al. (2020)	
	GEN1046	Bispecific antibody (PD-L1 xCD137)	I/II (FIH 2019)	• DCR 65.6% Muik et al. (2022)	NCT03917381
TNFRSF18 (GITR)	TRX-518	IgG1	I/II (FIH 2010)	• No DLT (0.0001 to 8 mg/kg)	NCT01239134 NCT02628574
				• Monotherapy: ORR 0% Koon et al. (2016)	
	MK-4166	IgG1	I (FIH 2014)	• No MTD reached (0.0015-900 mg)	NCT02132754
				• Monotherapy: ORR 0% Papadopoulos et al. (2019)	
	MK-1248	IgG4	I/II (FIH 2015)	• No DLT up to 170 mg	NCT02553499
				• Monotherapy; ORR 0%	
				• MK-1248/pembrolizumab; ORR 18% Geva et al. (2020)	
	BMS-986156	IgG1	I/II (FIH 2015)	• No DLT (10–800 mg)	NCT02598960 NCT04021043
				• Monotherapy: ORR 0%	
				• BMS-986156/nivolumab: ORR 2.7%–14.3% Heinhuis et al. (2020)	
	Efgivanermin alfa (MEDI 1873)	IgG1	I (FIH 2015)	• No MTD reached up to 750 mg	NCT02583165
				• Monotherapy; ORR 0% Balmanoukian et al. (2020)	

Although showing promising antitumor activities in preclinical tests, the clinical performance of TNFRSF agonists remains unsatisfactory. Dose-limiting toxicity or insufficient agonistic potency has been hampering the clinical development. For example, selicrelumab (CP-870893) and urelumab (BMS-663513) are two well-studied TNFRSF potent agonistic monoclonal antibodies undergone in clinical trials. Selicrelumab was an anti-CD40 agonistic antibody developed as human IgG2 showing potent activity without cross-linking. Urelumab was the first clinical strong anti-CD137 agonist developed as human IgG4 capable of binding to FcγRs and cross-linking, yielding a super agonistic antibody *in vivo*. Both agents showed treatment-related adverse events such as liver toxicity or cytokine release syndrome at low doses had maximal tolerated dose (MTD) at 0.2–0.3 mg/kg, which severely limited their full clinical evaluation.

Various efforts are being applied to optimize TNFRSF agonistic antibodies to overcome toxicity by improving their local agonism. Fc-engineering of anti-CD40 has been tested to enhance efficacy in the case of sotigalimab (APX005M), which uses IgG1 with S267E variation that has high affinity for FcγRIIB (Filbert et al., 2021). Newer agonistic antibodies leveraging FcγRIIB cross-linking with IgG4 such as ADG106 or CTX-471, or with designed IgG Fc mutant selective for binding to FcγRIIB such as LVGN6051 or LVGN7409, have also entered clinical development. Additionally, bi-specific antibodies have been developed by engineering an additional binding site to tumor antigen, such as HER2, fibroblast activation protein (FAP), or PD-L1 for targeted agonism towards tumors (Table 2). It remains to be seen if these attempts will resolve the clinical challenges for TNFRSF agonistic antibodies.

The rich literature on TNFRSF agonistic antibody development provides essential and excellent learnings for continued improvement in the approaches to optimize the design and development of TNFRSF agonistic antibodies. In this review, we focus on monoclonal antibody agonists with regular IgG structure.

## 4 Mechanism of Action of TNFRSF Agonistic Antibodies

### 4.1 Background on IgG and FcγR

#### 4.1.1 Human and Mouse Immunoglobulins

Among the five classes of human immunoglobulins (IgA, IgD, IgE, IgG, and IgM), IgG is most frequently chosen for therapeutic antibody development (Table 2). Antibodies possess regions that recognize antigens, the fragment antigen binding (Fab) region, and the effector function region, the fragment crystallizable (Fc) region that recognize Fc receptors and complement proteins. The second constant domain (CH2) in Fc and the first constant domain (CH1) in Fab are linked *via* a hinge. The hinge contains disulfide bonds from conserved cysteine residues that stabilize the structure of IgG, while allowing Fab and Fc conformational flexibility.

There are four IgG subclasses in human known as IgG1, IgG2, IgG3, and IgG4, and in mouse named as IgG1, IgG2a, IgG2b, and IgG3. They share more than 95% homology in the amino acid sequences of the Fc regions but show major differences in the amino acid composition and structure of the hinge region. IgGs participate in many important immune protection functions including the clearance of pathogens and toxins, and lysis and removal of infected or malignant cells. Detection and presentation of abnormal antigens are often the first steps for immune protection. This process involves the cellular uptake and intracellular processing of those antigens assisted by IgGs and FcγRs expressed on immune cells including antigen presenting cells (APCs).

#### 4.1.2 Human and Mouse Fc Gamma Receptors

**FIGURE 3:**
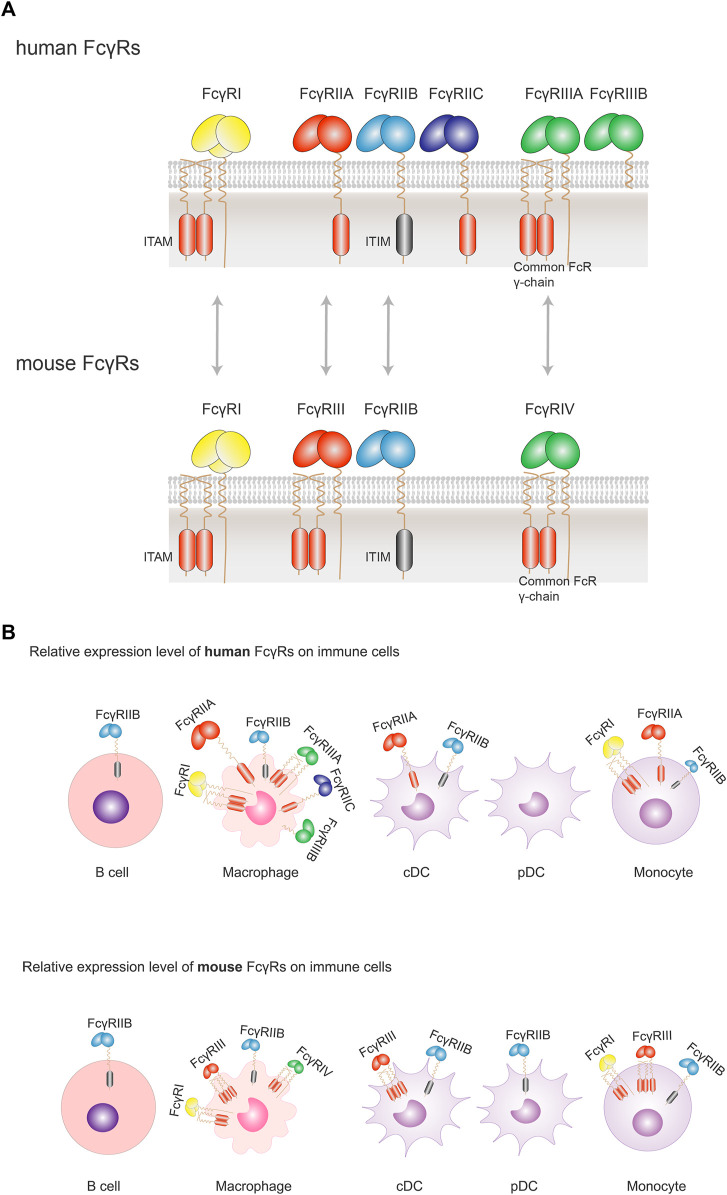
Orthologous pairs of FcγRs between human and mouse and their cellular expression. **(A)** In human, three groups of FcγRs have been described: FcγRI, FcγRIIA/B/C, FcγRIIIA/B. Orthologous pairs identified in mouse include human FcγRI and mouse FcγRI, human FcγRIIA and mouse FcγRIII, human FcγRIIB and Mouse FcγRIIB, and human FcγRIIIA and mouse FcγRIV. All activating FcγRs, except FcγRIIIB, are associated with an immunoreceptor tyrosine-based activation motif (ITAM) either in the intracellular domain (FcγRIIA and FcγRIIC) or associated with the common FcRγ chain (FcγRI and FcγRIIIA). There is only one inhibitory FcγR (FcγRIIB) in human or mouse. FcγRIIB contains an immunoreceptor tyrosine-based inhibitory motif (ITIM) in the intracellular domain. **(B)** FcγRs are expressed solely or simultaneously at the membrane of the various immune cells. FcγRI, FcγRIIA/B and FcγRIIIA are found on macrophages; FcγRIIA/B on conventional DC (cDC), and FcγRIIB (the sole FcγR expressed) on B cells. When co-expressed, activating FcγRs generally expressed more abundantly with respect to the inhibitory FcγRIIB. Despite a similar expression profile of FcγRs in human and mouse, there are some differences between the two species: FcγRIIB is not expressed on human pDCs, but on mouse pDCs; while co-expressed on the same cells, the inhibitory FcγRIIB is relatively less than activating FcγRs in human, which is not so apparent in mouse. Size of symbols of FcγRs is drawn to reflect their relative expression levels.

There are three major groups of human activating FcγRs (including FcγRI, FcγRIIA, FcγRIIC, FcγRIIIA, and FcγRIIIB). All activating FcγRs, except FcγRIIIB, are associated with an immunoreceptor tyrosine-based activation motif (ITAM) either in the intracellular domain (FcγRIIA and FcγRIIC) or associated with the common FcRγ chain (FcγRI and FcγRIIIA) (Vidarsson and van de Winkel, 1998; Bruhns, Iannascoli et al. 2009; Bruhns and Jönsson 2015). Similarly, there are three activating FcγRs in mouse (FcγRI, FcγRIII, and FcγRIV) with an ITAM motif. In contrast, there is only one inhibitory FcγR (FcγRIIB) in humans or the mouse. FcγRIIB contains an immunoreceptor tyrosine-based inhibitory motif (ITIM) in the intracellular domain (Nimmerjahn et al. 2005; Hogarth and Pietersz, 2012) (Figure 3A).

Following the binding to antigen-IgG immune complexes (ICs), ITAM of activating FcγRs is phosphorylated by Src kinases and induces downstream signaling, leading to various immune responses. ITAM-mediated functions include phagocytosis, degranulation, antibody-dependent cellular cytotoxicity (ADCC), cytokine, lipid mediator and superoxide production. In contrast, in the case of the inhibitory FcγRIIB engagement, the downstream signal transduction mediated *via* ITIM results in anti-inflammatory activities. As the only inhibitory FcγR, FcγRIIB plays an important role in preventing exaggerated auto-immunity and maintaining homeostatic immunity to disease prevalence and severity.

#### 4.1.3 FcγR Expression, Affinity and Function Profile

In humans, FcγRs are widely expressed in hematopoietic cells. B cells solely express FcγRIIB. Monocytes express FcγRI and FcγRIIA/B. Both monocyte-derived DCs and DCs from blood, but not plasmacytoid DCs (pDCs), express primarily FcγRIIA/FcγRIIB. Macrophages express all classes of FcγRs. It is worth noting that the activating FcγRs are generally more abundantly expressed than FcγRIIB. FcγRI is the only high-affinity receptor, and is able to bind to monomeric IgG. In contrast, FcγRIIA/B/C and FcγRIIIA/B are low-affinity receptors (Table 3), and therefore binding to FcγRII/IIIs require high-avidity of multivalent antigen-IgGs immune complexes complex (IC). Once antigens are recognized and bound by IgG-Fab, the resulting immune complexes (ICs) enable the Fc portion of the multivalent ICs to bind to low affinity FcγRs, leading to the clustering of multi-homo or hetero FcγRs. The clustering of FcγRs is important for their cytoplasmic signaling activation both for activating FcγRs and inhibitory FcγRIIB (Junker et al., 2020).

**TABLE 3:** Affinity in KD (μM) of human and mouse IgG subclasses for FcγRs.

FcγR		Human			Mouse				
IgG		hFcγRIIB	hFcγRI	^a^hFcγRIIA	hFcγRIIIA	mFcγRIIB	mFcγRI	^a^mFcγRIII	mFcγRIV
		Inhibitory	Activating	Activating	Activating	Inhibitory	Activating	Activating	Activating
Human	^b^hIgG1(h1)	3.01	0.0052	1.16	6.7	1.1	0.035	9.3	0.28
	^c^hIgG2(h2)	500	—	2.2	14	7.9	—	9.7	—
	hIgG4(h4)	50	0.029	5.9	4	11	0.19	21	26
Mouse	^b^mIgG2a(m2a)	N/A	N/A	N/A	N/A	2.5	0.0063	1.4	0.034
	^c^mIgG1(m1)	N/A	N/A	N/A	N/A	0.303	—	3.3	—
	mIgG2b(m2b)	N/A	N/A	N/A	N/A	0.45	—	1.7	0.059

—, no binding detected; N/A, not available.

a hFcγRIIa/mFcγRIII is identified to be orthologous pair between human and mouse; bhIgG1/mIgG2a and chIgG2/mIgG1 are proposed to be functional homologues

Since activating and inhibitory FcγRs are often co-expressed on the membrane of the same cell, it is the relative abundance of these FcγRs and the strength of the IgG-FcγR interaction that ultimately determines the cellular outcomes. The immune suppressive role of FcγRIIB might explicate its more modest expression profile than that of activating FcγRs. The rarer membrane presence of FcγRIIB suggests a higher binding or activation threshold, for example, requiring a higher valency of multivalent antigens-IgGs-FcγRs complex (Lehmann et al., 2017). Therefore, FcγRIIB won’t be activated too early to disturb the protection functions of the activating FcγRs during infection for example.

Interestingly, besides its immune suppressive function, FcγRIIB was demonstrated to help clustering of immune complexes, as knockout of FcγRIIB resulted in formation of smaller endosomes. In the studies on the phagocytosis of antibody coated nanoparticles, a role for FcγRIIB was identified for the aggregation of multivalent antibody bound nanoparticles. We speculate this clustering ability of FcγRIIB, which facilitates the aggregation of bound antigens-IgGs complexes in the case of phagocytosis, may contribute to the activity of TNFRSF agonistic antibodies via cross-linking, which will be discussed in detail later.

#### 4.1.4 Comparison of IgG/FcγR Interactions in Human and Mouse

The high affinity receptor FcγRI and the sole inhibitory FcγRIIB are highly conserved between human and mouse. Human FcγRIIA and mouse FcγRIII are identified to be orthologous receptors, since they share similarities in terms of genomic localization and sequence in the extracellular portion (Nimmerjahn and Ravetch, 2006, Hirano et al., 2007) (Figure 3A). In mouse, FcγRs show similar expression profile as their human orthologous pairs, except that the expression of FcγRIIB is slightly more abundant and broader among various immune cells than in human (Junker, Gordon et al. 2020) (Figure 3B).

Besides the heterogeneous expression of FcγRs, additional complexity of IgGs- FcγRs system was provided by different IgG subclasses, IgG1, IgG2, IgG3, and IgG4, with distinct binding affinities for different FcγRs (Table 3). Human IgG1 appears to be a functional homolog of mouse IgG2a, and human IgG2 for mouse IgG1. There are some nuanced differences between human and mouse, including the fact that human IgG2 binding affinity of human FcγRIIB is much lower than its orthologous mouse IgG1 to mouse FcγRIIB (Rosales and Uribe-Querol, 2013; Dekkers et al., 2017; Rosales, 2017). Together, that could explain the similarity of those homolog IgGs and FcγRs in terms of their expression profile and binding affinities.

In summary, high similarity is found between human and mouse IgG-FcγR systems system but accompanied with some exceptions. Therefore, cautions should be taken when transferring the data obtained in mouse models into the human system.

Human IgG1 and IgG4 preferably bind to FcγRI, whereas IgG2 fails to bind FcγRI. FcγRI is the only high-affinity IgG receptor. FcγRIIB has a relatively lower affinity for IgG1, IgG2 and IgG4 than FcγRIIA.

Murine IgG2a binds to FcγRI with significantly higher affinity than to other FcγRs, whereas neither IgG1 nor IgG2b binds to FcγRI. Murine IgG1 has a higher binding affinity to FcγRIIB than IgG2a.

Human IgGs bind to mouse FcgγR with similar binding ability to those for human ortholog receptors, with relative affinities IgG1>IgG4>IgG2 and FcγRI>>FcγRIV>FcγRIII>FcγRIIb. However, there are some subtle differences including but not limited to that human IgG2 has a higher binding affinity to mouse FcγRIIB than human FcγRIIB.

### 4.2 Structural Determinants of Agonistic Antibodies that Mimic TNFSF Ligand to Facilitate the Oligomerization and Activation of TNFRSF Receptors

TNFSF ligands naturally exist on cell membrane as trimers or dimers, which in turn can facilitate the formation of TNFRSF trimer and secondary clustering of oligomers leading to receptor activation. Monoclonal antibodies have shown agonistic activity towards TNFRSF members and been evaluated in the clinic as immune co-stimulation agents for cancer therapy as introduced above (Table 2). One key question is how agonistic antibodies mimic TNFSF ligand to facilitate the oligomerization of the receptors to trigger the downstream signaling cascades. The answer lies within the structural features of the IgG molecule, i.e., its flexible and multiple functional domains consist of bivalent antigen binding Fab arms, conformation determining hinge, and Fc dimer interacting with Fc receptors present on immune cells. It is understood that TNFRSF agonistic antibodies provoke the clustering of TNFRSF in the following ways: 1) bivalent Fab arms with high affinity binding to TNFRSF dimers/trimers; 2) proper hinge conferred by differential disulfide bonds supporting TNFRSF conformational changes for productive receptor clustering and activation; and, 3) Fc binding to membrane bound FcγRs which provide an “immobilized” scaffold to facilitate the aggregation of Fab bound TNFRSF, i.e., cross-linking. Each of these structural determinants individually and collectively attune the agonistic activity of the antibody *in vitro* and *in vivo*, with the Fc-FcγR interaction being the dominant factor.

#### 4.2.1 Cross-Linking Mediated by Fc and Fc Gamma Receptors

##### 4.2.1.1 TNFRSF Agonistic Antibodies Showing Dependence on FcγRIIB

The first demonstration of TNFRSF agonism dependency on FcγRIIB was a study focused on the development anti-Fas (TNFRSF6, DR2 or CD95) agonistic antibodies. *In vitro* studies showed that anti-Fas agonistic mAb Jo2 mediated apoptosis was enhanced by FcγRIIB, but not FcγRI and FcγRIII, on neighboring macrophages (Xu et al. 2003).

Agonistic antibodies targeting DR5, another TNFRSF death receptor, also displayed activities against a variety of tumors. Li et al. reported that the *in vivo* apoptotic and antitumor activities of these antibodies have an absolute requirement for FcγRIIB. Furthermore, enhancing FcγRIIB engagement *via* IgG1 S267E variant increased apoptotic and antitumor potency (Li and Ravetch, 2012).

Furthermore, agonist-targeted cells did not need to express FcγRIIB by themselves and FcγRIIB expression on bystander cells was sufficient, as demonstrated by the example that co-culture with CD40^−/−^ (FcγRIIB^+/+^) B cells allowed anti-CD40 3/23 agonistic mAb to promote proliferation of FcγRIIB^−/−^ (CD40^+/+^) B cells. This experiment also suggested that FcγRIIB downstream inhibitory signaling pathways were not required for promoting the agonistic antibody activity, as cross-linking mediated by extracellular part of FcγRIIB without intracellular domain is adequate for TNFRSF agonistic co-stimulation activities. More importantly, FcγRIIB has shown to be the only required FcγR for the antitumor activities *in vivo* (Li and Ravetch, 2013; White et al., 2013; White et al., 2014).

Besides DR2, DR5 and CD40, FcγRIIB-dependent agonism was also demonstrated for antibodies targeting CD137 and OX40 by exploiting FcγR deficient animals and/or antibody variants with defective FcγR binding.

Qi et al. evaluated the antitumor efficacy of two anti-CD137 agonistic antibodies with mouse IgG1 isotype, 3H3 with strong agonistic activity and LOB12.3 with weak agonistic activity *in vitro*. Both antibodies showed robust antitumor activity *in vivo*. However, the antitumor activity of LOB12.3-mIgG1 is significantly reduced in Fcgr2b−/− (FcγRIIB deficient) mice, while 3H3-mIgG1 showed a weaker but significant antitumor activity in Fcgr2b−/− mice. In Fcer1g−/− (lacking the common g chain, deficient in FcγRI, FcγRIII, and FcγRIV) and Fcgr3^−/−^ (FcγRIII deficient) mice, both LOB12.3-mIgG1 and 3H3-mIgG1 showed potent anti-tumor activity like in wild type mice. Taken together, these data indicate that FcγRIIB but not the activating FcγRI, FcγRIII, or FcγRIV is required for antitumor activity of agonistic anti-CD137 antibodies *in vivo*.

Ho et al. identified a new class of CD137 agonist mAbs which showed cross-linking-dependent T-cell co-stimulation activity *in vitro* (Ho et al., 2020). During further *in vivo* experiments, when different mouse IgGs subclasses were compared, IgG2a had stronger antitumor activity than the IgG2aDANA variant (D265A-N297A, mutant that eliminates FcγR binding), and both murine IgG1 and IgG2 consistently showed strong efficacy. Because both murine IgG1 and IgG2 engage FcγRIIB and III, Ho et al. evaluated which FcγR was critical for the bioactivity of CD137 mAbs. They demonstrated that antitumor efficacy was maintained in FcγRIII-deficient mice but diminished in FcγRIIB-deficient mice, suggesting the critical role for FcγRIIB to provide cross-linking and to induce antitumor activity *in vivo*.

The role of FcγRIIB was also demonstrated for anti-OX40 agonist. Campos Carrascosa, van Beek et al. used lymphocytes from resected tumor, tumor-free (TF) tissue and peripheral blood mononuclear cells (PBMC) of 96 patients with hepatocellular and colorectal cancers to test the *in vitro* T-cell agonistic activity of OX40-targeting antibodies (Campos Carrascosa et al., 2020). They showed that, in contrast to a clinical candidate anti-OX40 IgG1, treatment with an Fc-engineered anti-OX40 IgG1 v12 variant (E223D, G237D, H268D, P271G, Y296D, and A330R, selectively enhanced FcγRIIB binding affinity), stimulated *in vitro* CD4 and CD8 TIL expansion and shifted their transcriptional landscape toward a pro-survival, inflammatory and chemotactic profile. They also proved that the activity of anti-OX40 IgG1 v12 was dependent on FcγRIIB engagement.

Zhang et al. generated four anti-OX40 agonistic mAbs in mIgG1 and then evaluated their ability to mediate OX40 agonism using reporter cell line in the absence or presence of the mouse B lymphoma cell line A20, which expresses only mouse FcγRIIB (mFcγRIIB). They found that all four mAbs mIgG1 demonstrated the agonistic activity upon mFcγRIIB crosslinking (Zhang et al., 2019). In contrast, all four mAbs mIgG1 were fairly inactive in the absence of A20 cells or in the presence of A20 cells incubated with an anti-mFcγRIIB blocking antibody prior to performing the reporter assays. None of the four mIgG1 D265A variants, which abrogate binding to all mFcγRs, was able to activate OX40.

FcγRIIB was shown to be able to boost the agonism of the engineered anti-OX40 mAb IgG1 with mutations T437R and K248E, that facilitated antibody multimerization upon binding to antigens on cell surface (Zhang et al., 2017). For the note, T437R is a mutation predicted to facilitate the dimerization of CH3 domains, and K248E mutation is predicted to facilitate multimerization by destabilizing the intramolecular CH2:CH3 interface. The combination of the two mutations is predicted to facilitate antibody multimerization upon binding to antigens on cell surface.

These studies demonstrated that there is a positive correlation between the activity of many TNFRSF agonistic antibodies and the capacity of their Fc binding to the inhibitory FcγRIIB.

Conclusive evidence for the essential role of FcγRIIB for certain agonistic antibodies of TNFRSF came from studies using genetic mouse models of Fcγ receptor knockouts. Absolute requirement of FcγRIIB but not activating Fcγ receptors was demonstrated in above mentioned studies for DR2 (Xu et al., 2003), DR5 (Li and Ravetch, 2012), CD40 (Li and Ravetch, 2011; White et al., 2011; Wilson et al., 2011; Dahan et al., 2016), and CD137 (Qi et al., 2019; Ho et al., 2020).

##### 4.2.1.2 Similarities of the TNFRSF Secondary Clustering Mediated by Membrane- Bound Ligands and by Agonistic Antibodies Dependent on FcγRIIB Cross-Linking

**FIGURE 4:**
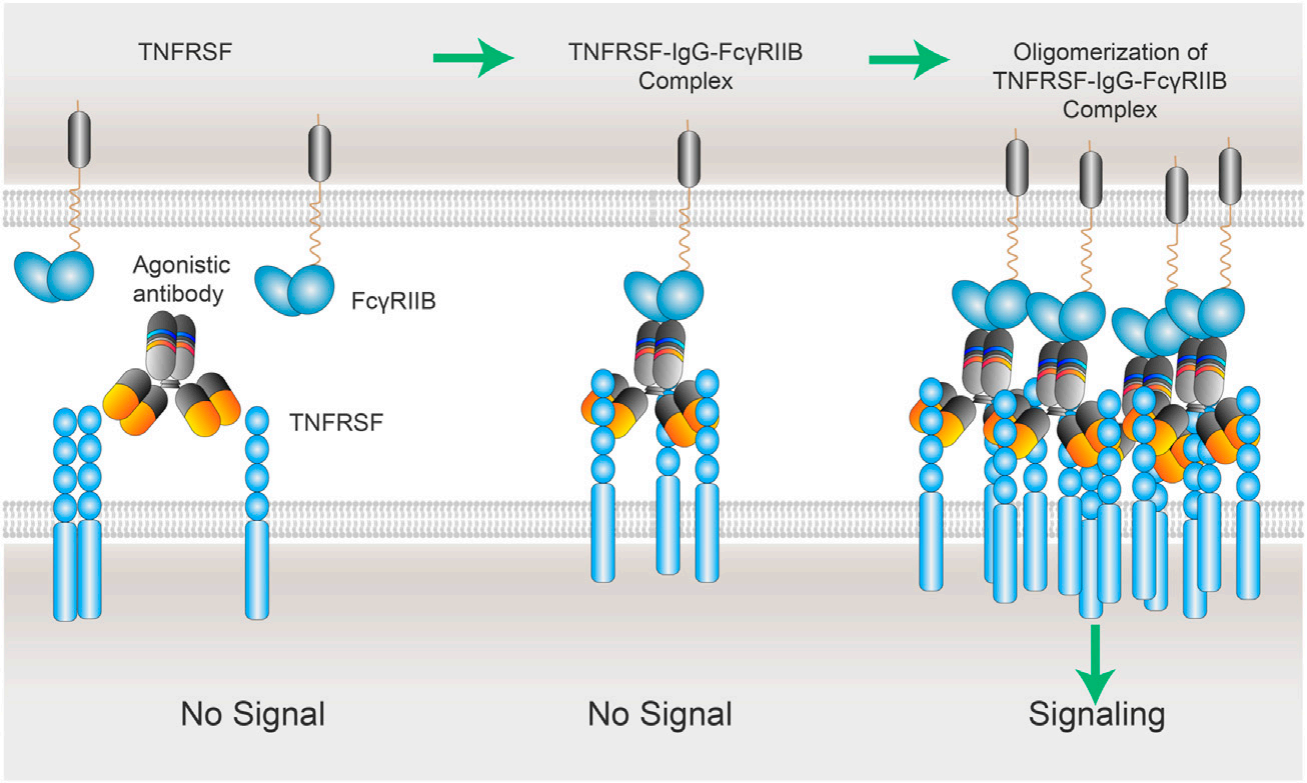
xLinkAb working model: the role of FcγRIIB in the oligomerization of TNFSFR-IgG-FcγRIIB complexes. xLinkAb TNFRSF agonistic antibody Fab arms binding to TNFRSF is insufficient to cluster and activate TNFRSF target; xLinkAb IgG-TNFRSF complex engages FcγRIIB with its engineered Fc selectively for FcγRIIB binding, leading to the formation of oligomic TNFRSF-IgG- FcγRIIB complexes and clustering of TNFRSF and signaling activation. The initial step should be IgG-Fab binding to TNFRSF, which is followed by TNFRSF-IgG-Fc binding to FcγRIIB forming TNFRSF-IgG-FcγRIIB complex and secondary clustering of TNFRSF. Activation of TNFRSF signaling depends on the formation of the multivalent TNFRSFs-IgGs-FcγRIIBs complexes and TNFRSF super-clustering.

In a study to determine if FcγR-dependent agonism is applicable for all the TNFRSF members, a coincidence was identified between the TNFRSF whose activation depended on secondary clustering mediated by membrane- bound ligands and those TNFRSF whose agonistic antibodies required the presence of FcγRIIB (Medler et al., 2019; Kucka and Wajant, 2020). Medler, Nelke et al. analyzed a panel of ∼30 murine antibodies recognizing more than 10 members of the TNFRSF for their capacity to elicit TNFRSF receptor activation. TNFRSF receptor responsive target cells were in co-cultured with empty vector (EV) control or murine FcγRIIB plasmid- transfected HEK293 cells IL8 induction was measured to detect TNFRSF receptor activation. With exception of one antibody against GITR, all other antibodies, recognizing CD27, OX40, 4-1BB, TNFR2, CD40, CD95, TRAILR1, TRAILR2 or Fn14, displayed no or only modest agonistic activity when TNFRSF receptor responsive target cells co-cultured with EV control. But they readily converted to potent receptor agonists in the presence of murine FcγRIIB-expressing cells. The coincidence is that those TNFRSF are only activated by their membrane- bound ligands but not by the soluble ligands. The data suggest that the threshold of activation is more exigent for those TNFRSF whose activation needs the oligomeric clustering mediated by the membrane- bound scaffold. This “immobilized” support is offered either by membrane- bound ligands or IgGs binding to FcγRIIB. It is tempting to postulate that the dependency on FcγRIIB is based on a superior clustering ability of membrane- bound FcγRIIB bridged by IgGs. In a similar model as membrane bound ligands, those FcγRIIB -“immobilized” IgGs facilitate the clustering of their Fab-bound TNFRSF, resulted in the aggregation of FcγRIIBs-IgGs-TNFRSFs complexes, and eventually TNFRSF cytoplasmic activation (Figure 4).

##### 4.2.1 3 Cross-Linking by Other FcγRs

In addition to FcγRIIB, other Fcγ receptors can cross-link antibodies to enhance their agonistic activity for TNFRSF *in vitro*. This is consistent with the working model of scaffold effect, which can be achieved by secondary antibodies or by simple physical immobilization on microtiter plate surface. However, FcγRs other than FcγRIIB do not contribute to the antitumor efficacy *in vivo* in mouse models for the agonistic antibodies whose activity depends on cross-linking. For example, the effect of FcγRIIB on anti-CD40 agonistic activities was investigated both *in vitro* and *in vivo*. *In vitro* studies showed that both mouse FcγRIIB and FcγRIII could promote cross-linking dependent agonistic activity and stimulated proliferation of B cell activity of a rat anti-mouse CD40 mAb, 3/23, in the form of mouse IgG1 (White et al., 2011). *In vivo* studies, however, demonstrated that the antibody was efficacious in mice with knockout of activating Fcγ receptors. The antibody lost activity only when FcγRIIB was absent (White et al., 2011).

Activating Fcγ receptors are not only dispensable for the antitumor efficacy of agonistic antibodies, but also can be detrimental due to the Fc effector function resulting in elimination of active immune cells by ADCC or ADCP. When tested in hCD40Tg mice, two anti-CD40 human agonistic antibodies ChiLob 7/4 IgG2 and SGN40 IgG2 (very low binding affinity to all FcγRs) stimulated strong expansion of T cells, whereas their corresponding IgG1s (high binding affinity to activating FcγRs) were significantly less active (Yu et al., 2018). White et al. showed that mouse IgG2a antibody (equivalent to human IgG1 capable of ADCC and ADCP) was less effective than mouse IgG1 antibody (Fc effector null). Qi et al. also showed that mouse IgG2a antibody against 4-1BB LOB12.3 had lower antitumor activity than the mouse IgG1 version suggesting the negative impact of effector function.

It is now well accepted that Fc binding to activating Fcγ receptors should be avoided in therapeutic co-stimulation TNFRSF agonistic antibodies.

#### 4.2.2 Fab, Epitope and Ligand Competition

The binding epitope of TNFRSF target are key factors contributing to the intrinsic agonistic activities of the antibody. A correlation was found between the binding epitope and agonistic activity, and the strongest agonists were suggested to be those antibodies binding to the very N-terminus of TNFRSF at CRD-1 which is likely maximally exposed on the surface of the cell, or because of non-competition with natural ligand (Chin et al., 2018). For example, antibodies that bind to CRD1 of CD40 (ChiLob 7/4, SGN40, CP870,893 and Lob 7/2 in same IgG isotype) are stronger agonists, and the CRD2-4 binding mAbs are less agonistic (Yu et al., 2018). However, it is worth noting that ChiLob 7/4 and SGN40, although both anti-CD40 mAbs binding to CRD1 (Vonderheide et al., 2007; Hussein et al. 2010; Johnson et al. 2015), showed much lower agonistic activity than CP870,893 (Yu et al., 2018), suggesting that binding to CRD1 itself doesn’t necessarily guarantee strong agonistic activities.

CD137 agonistic antibody urelumab and utomilumab provided another example of epitope difference (Chin et al., 2018). Urelumab binds CRD1 of CD137 and exhibits strong agonistic activity, while utomilumab binds to CRD3/4 and shows weak activity. The agonistic activity difference was confirmed when these two antibodies were expressed in the same IgG isotype in our lab.

However, the correlation between CRD1 binding and strong agonistic activity is not observed in a study on anti-OX40 agonists. Zhang, Tu et al. generated and characterized a panel of anti-mOX40 mAbs targeting the four mOX40 CRDs and evaluated their *in vitro* and *in vivo* agonistic activities along with OX86, a benchmark anti-OX40 agonistic mAb. In striking contrast to agonistic mAbs targeting CD40 and CD137, they found that agonistic mAbs targeting OX40 do not require CRD1 binding and non-ligand blocking for *in vivo* agonistic function. While all four mAbs enhanced the *in vivo* immune response, CRD2- and CRD4-binding instead of CRD1-binding anti-mOX40 mAbs showed the highest levels of *in vivo* agonistic activities in terms of stimulating CD8 Teff cell expansion and anti-tumor activity, highlighting that it remains unknown how the binding epitope affects the intrinsic agonistic potential of mAbs (Zhang et al., 2019).

The possible competition between the agonistic antibodies and endogenous ligands might influence the outcome of TNFRSF signaling activation. Either CD137L or CD40L binds to CRD2-3 of its cognate receptor, and the CRD2-3 binding agonistic antibodies can at least partially compete with the ligand for receptor binding. It is not yet totally understood how this potential competition affects the therapeutic outcome of TNFRSF agonistic antibodies.

The effect of Fab sequence on agonistic activity might be explained by steric acceptability of the membrane-bound receptor. Certain TNFRSF agonistic antibodies can have sufficiently strong agonistic activity without Fc-FcγR cross-linking. This intrinsic agonistic activity could still be affected by Fc-FcγRIIB interaction (vide supra), leading to distinct overall agonistic outcome in the body that will be discussed in more details (vide infra 4.2.4).

#### 4.2.3 Hinge and IgG Flexibility

The flexibility of IgG is also suggested to play a role antibody agonistic activity. IgG2 and more precisely its unique arrangement of disulfide bonds in the hinge region, has been shown to impart superior agonistic activities (White et al. 2015; Yu et al. 2020). Yu et al. dissected the mechanism of an IgG2-mediated, FcγR-independent, agonism. They noted that when the anti-CD40 mAbs were engrafted onto IgG2, the antibodies showed enhanced FcγR-independent agonistic activity. IgG2 is unique among human IgG in its ability to “shuffle” disulfide bonds in its CH1 and hinge regions, resulting in a range of isoforms. The molecule is believed to be synthesized in its “h2A” form having a classical IgG flexible “Y” conformation wherein the heavy chain (HC) Cys127 in CH1 is linked to Cys214 in the light chain (LC). This “h2A” form gradually converted to the “h2B” form in the blood, adopting a more compact shape with the Fab’ arms held in close proximity to the hinge, wherein HC Cys127 form disulfide bonds with the HC hinge Cys232 and LC Cys214 with HC hinge Cys233 (Martinez et al., 2008; Allen et al., 2009). The mutagenesis was used to produce “locked” h2A-like forms (HC C232S or C233S mutation) and h2B-like forms (HC C127S mutant) (Martinez et al., 2008, Allen et al., 2009). White, Chan et al. showed that anti-CD40 agonist ChiLob 7/4 in h2B-like form had increased activity relative to native ChiLob 7/4 h2 to stimulate the proliferation of hCD40 Tg B cells. In contrast, ChiLob 7/4 in h2A-like form totally lost that agonistic activity. The data suggested that the FcγR-independent agonistic activity of h2 is contingent upon the precise conformation of disulfide bonds in its hinge and CH1 domains, and specifically on its ability to adopt the more compact h2B form (White et al., 2015). Some TNFRSF agonistic antibodies were demonstrated to possess autonomous agonistic activity without secondary cross-linking by FcγRIIB (Richman and Vonderheide, 2014). Such antibodies are known as FcγRIIB independent and have sufficient activity *in vivo* in the absence of FcγRIIB cross-linking. Nevertheless, their activity can still be enhanced when Fc-FcγRIIB cross-linking is available.

Like Fab, the IgG hinge is just one of the structural determinants for antibody overall agonistic activity, which is the integrated sum of these individual components in our body where Fc-FcγR interaction occurs naturally and should be understood as a whole.

### 4.3 Fc-FcγRIIB Interaction as the Dominant Factor for Therapeutic Agonistic Activities of TNFRSF Antibodies

Physiological TNFRSF receptor activation is induced upon binding to cell-surface TNFSF ligand via cell-cell interaction, resulting in receptor-ligand trimer cross-linking and oligomerization. Clustering of receptors leads to productive docking of intracellular adaptor molecules and assembly of signaling machinery. Antibodies can activate TNFRSF because of their ability to cross-link receptor dimers or trimers on cell surface, but their activity differs widely due to a combination of factors including various Fab, hinge and Fc sequences. Each of the structural features of an antibody contributes to the agonistic activity and can be evaluated in experiments individually. However, the overall agonistic activity is an integration of all interactions in the microenvironment of the tissues. Based on the experimental data, we consider that Fc-FcγRIIB interaction is the dominant factor for therapeutic agonistic activities of TNFRSF antibodies.

#### 4.3.1 FcγRIIB-Dependent TNFRSF Agonistic Antibodies With High Therapeutic Index

Although the importance of FcγRIIB in enhancing agonistic activity of TNFRSF antibodies was recognized as early as 2003 (Xu et al., 2003), it was not until more recently that FcγRIIB-dependent CD137 agonistic antibodies were shown to have superior safety while retained strong antitumor activity (Qi et al., 2019, Ho et al., 2020). Liver toxicity and antitumor efficacy of anti-CD137 antibodies were investigated in preclinical mouse models. Both LOB12.3 and 3H3 antibodies showed similarly strong antitumor efficacy, but they exhibited distinct liver toxicity profiles; 3H3 significantly increased alanine transaminase (ALT) levels, whereas LOB12.3 had minimal impact. Besides elevation of ALT in serum, 3H3 but not LOB12.3 caused immune cell infiltration in the liver. The study revealed that anti-CD137 agonistic antibody-induced antitumor activity and liver toxicity could be separated. Further mechanism studies showed that 3H3 is a strong agonistic antibody in the absence of Fc-FcγRIIB cross-linking while LOB12.3 exhibits agonist activity only when Fc-FcγRIIB cross-linking is available. *In vivo*, 3H3 induced liver toxicity without Fc-FcγR cross-linking, either as Fc-null mutants or in FcγR knockout mice. In a prior mouse study, the non-FcγR cross-linking dependent 3H3 antibody was shown to induce multifocal mononuclear cell infiltrations in the liver. CD8 T cells, TNF-α, type I IFNs, and IFN-γ were essential for the induction of various aspects of pathology. Additionally, autoimmune mechanism was ruled out, while cytokine-induced inflammation was considered as the primary cause of liver toxicity (Niu et al., 2007).

Ho et al reported a new class of CD137 agonist monoclonal antibody with strong anti-tumor potency without significant transaminitis *in vivo*, which showed crosslinking-dependent T cell co-stimulation activity *in vitro* (Ho et al., 2020). Antitumor efficacy was maintained in Fc gamma receptor FcγRIII-deficient mice but diminished in FcγRIIB-deficient mice, confirming the critical role for FcγRIIB to provide cross-linking *in vivo*.

These landmark preclinical studies provide insights for discovery and clinical development of TNFRSF agonistic antibodies by exploiting the Fc-FcγRIIB cross-linking biology.

#### 4.3.2 The Role of FcγRIIB *in vivo*

The effect of FcγRIIB on anti-CD40 agonistic activities was investigated both *in vitro* and *in vivo*, and anti-CD40 agonism was demonstrated to be enhanced by either mouse FcγRIIB or FcγRIII *in vitro* while only by FcγRIIB *in vivo*.

*In vivo*, FcγRIIB is widely expressed in hematopoietic cells and often co-expressed with other activating FcγRs, more frequently with FcγRIIA. FcγRIIB generally shows a lower expression level than other FcγRs. One exception is on B cells where FcγRIIB is the only FcγR expressed (Junker et al., 2020). Firstly, it is tempting to speculate an important role of B cells in TNFRSF agonistic mAb activities *in vivo*. Indeed, B cells may concentrate at tumor margins or form tumor-associated immune complex structures such as tumor-associated tertiary lymphoid structures (TLS). In particular, the colocalization of B cells and T cells were reported in breast cancer, malignant melanoma and ovarian cancer (Milne et al. 2009; Ladányi et al. 2011; Nielsen et al. 2012; Iglesia et al. 2014; Sharonov et al., 2020). As mentioned above, FcγRIIB is expressed relatively highly on macrophages and DCs, and low on monocytes. We assume that FcγRIIB-dependent TNFRSF agonist could stimulate immune anti-tumor activities mediated *via* FcγRIIB expressed on the neighboring macrophages and DCs in tumor microenvironment (TME), with a limited effect on circulating monocytes because of FcγRIIB rareness, which resulted in an optimized efficacy and safety for cancer treatment.

Furthermore, the expression of FcγRIIB is not unchanged but dynamically influenced by cytokine exposure. It is reported that cytokines such as IL-10, IL-6 increase expression of FcγRIIB, while TNF-α and IFN-γ inhibit its expression (Anania et al., 2019).

For the majority of anti-CD40 agonistics in distinct mouse IgG format, mouse IgG1 format was demonstrated to promote an increase in CD8 T cell stimulation and antitumor activities, in contrast, mouse IgG2a was inactive. In light of the requirement for FcγRIIB in anti-CD40 agonistic activity, the difference between mouse isotypes can be explained by the approximately ten-fold higher affinity of IgG1 for FcγRIIB than that of IgG2a (Table 3).

For the TNFRSFs agonistic human IgG, the efficacy of the IgG1 (h1) and IgG2 (h2) isotypes of anti-CD40 are compared *in vivo* in the context of mouse FcγRs (mFcγR) or human FcγRs (hFcγR). In the context of mFcγR *in vivo* models, both h1 and h2 isotypes had adjuvant effects on T cell activation, and h2 resulted in a significantly higher T cell response as compared to h1 isotype of the same anti-CD40 clone. Particularly in the case of Lob 7/2, SGN40 and ChiLob 7/4. A remarkable difference between the agonistic activity of h2 and h1 is that the former is FcγR interaction independent and the latter is mFcγRIIB dependent (Richman and Vonderheide, 2014, White et al., 2015).

However, in a delicate study, a panel of humanized TNFRSF and FcγRs mouse models were generated to evaluate human agonistic IgGs in the human FcγR (hFcγR) background. For example, the CD40 humanized mice are crossed to the humanized FcγR mice. These hCD40/hFcγR mice recapitulate hCD40 and hFcγR expression patterns and levels in human, and hFcγR is functionally competent in the mouse background (Dahan et al., 2016). Those mice models were then used to test the agonistic activity of the anti-CD40 antibodies CP-870,893 and ChiLob 7/4 of various IgG subclass.

In the context of these hFcγR *in vivo* mouse models, h1 was the most active of the human isotypes, particularly in the case of Lob 7/2, SGN40, and ChiLob 7/4, where h1 showed agonism while h2 was less active. CP-870,893 h1 was shown to be unique in evoking very high agonistic activity, which was completely abolished when N297A mutation was introduced in Fc. An even lower activity was observed in deglycosylated form of h2 compared with wild-type h2. As N297A prevents binding to FcγRs and deglycosylated h2 has reduced binding affinity to FcγRIIB compared with h2 wild-type, it was suggested that FcγR-engagement is required for the agonistic activity of the anti-CD40 h1 and FcγRIIB was also involved in agonistic activity of anti-CD40 h2 (Dahan et al., 2016). Higher agonistic activity of h1 than that of h2 could be explained by the higher binding affinity of h1 to FcγRIIB than that of h2 (Table 3).

## 5 FcγRIIB Dependent TNFRSF Agonistic Antibodies Discovery and Development

### 5.1 Working Model of TNFRSF Agonistic Antibody Dependent on FcγRIIB Cross-Linking

#### 5.1.1 xLinkAb Model of FcγRIIB-Dependent and IgG-Bridged TNFRSF Clustering

Inspired by the studies on the relationship between the agonistic activities of TNFRSF antibodies and FcγRIIB dependency, we have proposed a cross-link antibody (xLinkAb) working model to optimize therapeutic antibodies by engineering Fc with selective FcγRIIB binding ability to match a desirable Fab to achieve tumor-targeted agonistic activity (Figure 4).

Although the binding to TNFRSF takes place in IgG-Fab region, the potential IgG-Fc binding to FcγRs expressed on surrounding cells has significant and dominant impact on the agonistic activities of IgG. More importantly, FcγRIIB-dependent CD137 agonistic antibodies have been shown to have superior safety while retained strong antitumor activity (Qi et al., 2019; Ho et al., 2020). One possible explanation for the dependency on FcγRIIB for TNFRSF agonistic antibodies is the common clustering ability shared by TNFRSF ligand (TNFSF) and FcγRIIB. Trimer of TNFRSF ligands triggers the formation of TNFSF_3_-TNFRSF_3_ complex, which is followed by oligomerization of two and more TNFSF_3_-TNFRSF_3_ complexes. Co-stimulation receptors including CD40, CD137, OX40, and GITR require those multi-trimeric structures to fully stimulate their downstream signaling cascades. In the case of TNFRSF agonistic antibodies, the initial step should be IgG-Fab binding to TNFRSF, which is then followed by TNFRSF-IgG-Fc binding to FcγRIIB forming TNFRSF-IgG-FcγRIIB complex and secondary clustering. Activation of TNFRSF signaling depends on the formation of the multivalent TNFRSFs-IgGs-FcγRIIBs complexes and TNFRSF super-clustering.

Analysis of single-cell RNA-seq (scRNA-seq) data and the literature reveals that FcγRIIB shows a unique expression profile on various immune cells enriched in the tumor microenvironment (TME). Firstly, emerging data indicate that intra-tumoral macrophages and dendritic cells, which are critical effectors underlying antibody induced antitumor immunity, express FcγRIIB at highest density (Arce Vargas et al., 2018). Secondly, as discussed earlier, FcγRIIB is the sole FcγR expressed on B cells which are also generally present in TME. In addition, moderate/absent expression of FcγRIIB on monocytes/pDC suggests its less abundance in the blood and normal tissues compared to TME. The cellular expression profile supports the observation that FcγRIIB cross-linking dependent agonistic antibodies could achieve acceptable therapeutic index.

The inhibitory FcγRIIB is low-affinity FcγR, which means it is unable to bind to circulating monomeric IgG. Its binding to IgGs is high-avidity driven thanks to multivalent antigen bound IgG structures. The aggregation of multivalent TNFRSF-IgG-FcγRIIB complexes might be mutually facilitated by Fab-bound TNFRSF clustering and Fc-bound FcγRIIB clustering (Figure 4).

Based on the xLinkAb model, FcγRIIB dependent TNFRSF agonistic antibodies would show optimal agonistic activity in a tumor microenvironment where both co-stimulation target TNFRSF and FcγRIIB are present on infiltrating immune cells and readily available for interaction. Because of the tumor-targeted agonism, FcγRIIB-dependent TNFRSF agonistic antibodies would be better tolerated due to diminished activity in circulation and normal tissues where FcγRIIB and TNFRSF rarely are present in sufficient density.

#### 5.1.2 Consideration and Uncertainty of FcγRIIB-Dependent Agonistic Antibodies

There are several important factors to be considered in engineering and optimizing FcγRIIB-dependent TNFRSF agonistic antibodies for cancer immunotherapy. Firstly, the antigen-binding Fab sequences must be extensively evaluated and only those with low apparent agonistic activity in the absence of cross-linking may be suitable. For example, urelumab (anti-CD137 agonist) and selicrelumab (anti-CD40 agonist) exhibit strong agonistic activity without secondary cross-linking as they recognize the most external CRD domain of either CD137 or CD40, and can efficiently cluster the receptor trimers with the bivalent Fab arms. The strong apparent agonistic activity may be sufficient to provide antitumor efficacy without Fc-FcγRIIB cross-linking. However, their agonistic activity could be further enhanced when the Fc-FcγRs interactions are engaged *in vivo.* Therefore, they could become super agonists in the body and thus are less tolerated. Additionally, the non-cross-linking dependent TNFRSF agonistic antibodies exhibit widely target agonism without modulation control and may cause on-target and off-tumor side effects. Therefore, the intrinsically strong agonistic antibodies are not desirable candidate for optimization to FcγRIIB dependent agonistic antibodies. On the other hand, not all intrinsically weak agonistic Fab sequences can be used for development of Fc-FcγRIIB cross-linking dependent agonistic antibodies without trial-and-error analysis. It would be ideal that the agonistic activity reaches a desired high level under Fc-FcγRIIB cross-linking conditions while remaining silent or low without cross-linking conditions. Limited by the expression profile of FcγRIIB mainly on B cells, macrophages and DCs accumulated in the tumor microenvironment, FcγRIIB-dependent TNFRSF antibodies are predicted to have a narrower scope of co-stimulating targeted cells and less systemic side effects.

Secondly, selection of hinge may be important. The apparent agonistic activity of a given Fab can change in different hinge contexts as discussed earlier. Thus, hinge can be used as an engineering tool in the optimization process.

Thirdly, Fc-FcγRIIB binding affinity and selectivity should be elaborately evaluated for each TNFRSF target and antibody. Native human Fc does not have a strict selectivity profile for Fc gamma receptors. FcγRIIB is a low affinity receptor for IgGs. There are successful examples in engineering more potent FcγRIIB binding mutants such as S267E (SE) and S267E/L328F (SELF) (Chu et al., 2008; Dahan et al., 2016), but selectivity optimization may be a challenging task. In addition, enhanced binding affinity to FcγRIIB could induce FcγRIIB ITIM activity, which might result in undesired immune effects. Further studies on the effect of FcγRIIB signaling is highly needed.

There is expression of FcγRIIB in normal tissues and especially in liver which may be a liability for liver toxicity. Therefore, Fc variants with high affinity to FcγRIIB may need to be avoided. It is critical to balance the Fab, hinge and Fc activity as whole both *in vitro* and *in vivo* settings.

### 5.2 First Generation Fc-Engineering to Enhance Binding Affinity to FcγRIIB

The initial Fc-engineering for TNFRSF agonistic antibodies focused on enhancing absolute binding affinity to FcγRIIB. The pioneering work was exemplified by APX005M (Filbert et al., 2021), which was engineered to introduce the S267E mutation in IgG1. APX005M strongly bound FcγRIIB (KD = 5.8 × 10^−8^ M), and required crosslinking for optimal agonistic activity. APX005M has advanced to Phase II clinical development (Table 2).

To improve CD40 agonistic antibodies, IgG1 Fc variants of CP-870,893 carrying the mutations S267E (SE) and S267E/L328F (SELF) were made and they showed 30- and 70-fold increased binding affinity to FcγRIIB, and resulted in increased ability to activate T cells and stronger antitumor activities (Dahan et al., 2016). Due to the high similarity of the extracellular part between FcγRIIA and FcγRIIB, the SE and SELF variations, resulting in increased binding affinity to both the activating FcγRIIA and the inhibitory FcγRIIB, thus a limited increase of the antitumor activity *in vivo* (Dahan et al., 2016).

Two additional Fc variants, G237D/P238D/P271G/A330R (V9) and G237D/P238D/H268D/P271G/A330R (V11), showed selective enhancement on FcγRIIB binding. CP-870,893 IgG1 with V9 variation (h1-V9) and CP-870,893 IgG1 with V11 variation (h1-V11) resulting in 32- and 97-fold increased binding affinity to hFcγRIIB, respectively, and about 3-fold decreased binding affinity to hFcγRIIA R131. Both h1-V9 and h1-V11 have significantly improved *in vivo* activity compared to the IgG2 subclass of CP-870,893 (h2). The CP-870,893 h1-V11 results in an increase in T cell activation and anti-tumor activity *in vivo* compared to h2 and h1-SELF. The increased agonism on T cell activation and anti-tumor activity was also observed in two other anti-CD40 mAbs CD40.1 (CRD2-3 binding) and CD40.2 (CRD1 binding) h1-V11 compared to their h1 or h1-SELF respectively (Dahan et al., 2016), suggesting that selective enhanced binding to FcγRIIB results in stronger agonistic activity and is rather a general rule than specific Fab clones. However, h1-V11 showed absolutely increased binding affinity to FcγRIIB from 3.0 × 10^−6^ M (K_D_) to 3.2 × 10^−8^ M (K_D_), similar to that for CD40 of 2.7 × 10^−8^ M (K_D_) (Dahan et al., 2016). Accompanied with an increased agonistic activity, there might be some bystander undesired effects. It was indeed observed that CP-870,893 h1-V11 caused the most significant decreases in body weight and platelet count after a single injection, compared with h2 or h1-SELF, and a similar phenomenon was observed for another anti-CD40 agonistic antibody CD40.2 (Dahan et al., 2016).

Several newer CD137 agonistic antibodies using IgG4 for FcγRIIB cross-linking have entered the clinic including ADG106 and CTX-471 (Table 2). Given that IgG4 binds to the high affinity FcγRI in addition to FcγRIIB and FcγRIIA, their *in vivo* activity may not solely depend on FcγRIIB and unwanted CD137 activation may affect their efficacy and safety profile. It is also unknown if these antibodies could activate CD137 as efficiently as the benchmark agonistic antibody urelumab.

### 5.3 Second Generation Fc-Engineering for FcγRIIb Selectivity on xLinkAb Platform

In our earlier study, we demonstrated for the first time that the antitumor efficacy and liver toxicity characteristics of anti-CD137 agonistic antibodies can be separated based on the agonistic ability and isotype. Proper combination of intrinsic agonistic strength and Fc-FcγRIIB cross-linking collectively determine the antitumor and liver toxicity property of TNFRSFs agonistic antibodies. The xLinkAb model and platform enabled the discovery of the second generation CD137 agonistic antibody LVGN6051 (Qi et al., 2019; Fu et al., 2021). LVGN6051 is a weak agonistic antibody which required FcγRIIB-mediated cross-linking for optimal agonistic activity. In CD137 activation reporter assay, LVGN6051 showed significantly enhanced co-stimulation ability when FcγRIIB was present. Importantly, LVGN6051 showed T cell co-stimulation ability comparable to a urelumab analog and superior to a utomilumab analog when FcγRIIB-expressing cells were present. Furthermore, LVGN6051 showed robust tumor control ability in a wide range of dosage. Most importantly, it did not induce liver toxicity while maintaining potent anti-tumor activity. LVGN6051 has shown an outstanding safety profile in the clinic with promising sign of antitumor activity (Qi et al., 2019; Fu et al., 2021).

LVGN7409 is a recombinant monoclonal antibody against CD40. The modified Fc fragment retains residual binding to FcγRIIB. LVGN7409 activates CD40 signaling in Fc-FcγRIIB cross-link dependent manner and thus operates optimally in a CD40 and FcγRIIB-enriched tumor microenvironment. LVGN7409 demonstrated more robust antitumor efficacy and superior safety profile than published CD40 agonist antibodies in preclinical models. Preliminary clinical safety and activity has been observed (Fu et al., 2022).

## 6 Conclusion

TNFRSF signaling stimulates the activation, proliferation of various immune cells, including myeloid cells and T cells, providing potential targets for cancer immunotherapy application. However, TNFRSF agonistic antibodies have shown limited success in the clinic, which may be due to limited receptor clustering-mediated signaling or associated dose-limiting toxicities of these early generation products. The studies investigating mechanisms and functions of various TNFRSF agonistic antibodies have identified an important role of FcγRIIB. FcγRIIB has been shown to mediate TNFRSF clustering bridged by TNFRSF antibodies. The agonistic activity of an antibody can be impacted by the Fab, hinge or Fc region, but the Fc-FcγRIIB interaction has been identified as the dominant determinant for the overall agonistic activity outcome (efficacy and toxicity). Strong agonistic antibodies without Fc cross-linking can be identified *in vitro*, and such antibodies would exhibit agonism upon target binding and become super-agonist when cross-linking of Fc-FcγRs is available. TNFRSF antibodies with weak or no detectable agonistic activity in the absence of Fc cross-linking can exhibit strong agonism when Fc-FcγRIIB is engaged.

Two important discoveries came from the studies of TNFRSF agonistic antibodies in mouse models. Firstly, only Fc-FcγRIIB cross-linking contributes antitumor immunity and efficacy although other FcγRs can cross-link TNFRSR-IgG to enhance agonism. Secondly, TNFRSF agonistic antibodies that rely on Fc-FcγRIIB cross-linking to show activity can induce antitumor efficacy without liver toxicity, indicating feasibility of selective agonism in tumor microenvironment. As the sole inhibitory Fcγ receptor, FcγRIIB has unique structural features and expression profile, which may contribute to these important observations. FcγRIIB is expressed exclusively on B cells that do not express other FcγRs, high on MDSC, widely on macrophages and DCs, and low on monocytes in the blood. Tumor tissues are rich in immune infiltrates including B cells, MDSC, macrophages and DC cells. Thus, FcγRIIB is present in higher density relative to normal tissues. The differential expression profile and level between tumor and normal tissue supports the separation of efficacy and toxicity of TNFRSF agonistic antibodies driven by Fc-FcγRIIB cross-linking.

Therefore, independent research teams from academic, biotech and pharmaceutical companies have been applying Fc engineering approaches to improve tumor-targeted TNFRSF clustering for efficacy and safety. In particular, the conditional TNFRSF agonistic antibodies with engineered Fc for enhanced affinity or selectivity for FcγRIIB, represents a new wave of therapeutic agents entering clinical development. FcγRIIB-dependent crosslinking of agonistic antibodies should be applicable for a majority, if not all, of TNFRSF members including CD40, CD137, OX40, GITR, and CD27. Furthermore, cross-linking dependency could be applied in developing TNFRSF bispecific or multi-specific antibodies, where FcγRIIB could be replaced or supplemented by a tumor antigen or immune target. Consequently, binding to these tumor-selective targets could result in the formation of multivalent tumor-targeted -antibodies-TNFRSFs complexes, which in turn cause the clustering of TNFRSF and activation of downstream signaling selectively in the tumor microenvironment.

Taken together, TNFRSF immune co-stimulation targets hold great potential to expand immunotherapy clinical benefits to more cancer patients. TNFRSF agonistic antibodies with regular IgG structure remain a desirable format to deliver clinical efficacy and safety with a mature manufacturing and commercial platform. It has become evident and important to explore Fc biology and Fc engineering, including our xLinkAb approaches guided by the principle of FcγRIIB cross-linking dependent agonism, to achieve breakthroughs in targeting TNFRSF for cancer immunotherapy.
